# Disease-Specific Mortality and Secondary Primary Cancer in Well-Differentiated Thyroid Cancer with Type 2 Diabetes Mellitus

**DOI:** 10.1371/journal.pone.0055179

**Published:** 2013-01-31

**Authors:** Szu-Tah Chen, Chuen Hsueh, Wen-Ko Chiou, Jen-Der Lin

**Affiliations:** 1 Department of Internal Medicine, Chang Gung Memorial Hospital, Chang Gung University, Taoyuan, Taiwan, Republic of China; 2 Department of Pathology, Chang Gung Memorial Hospital, Chang Gung University, Taoyuan, Taiwan, Republic of China; 3 Department of Industrial Design, Healthy Aging Research Center, Chang Gung University, Taoyuan, Taiwan, Republic of China; Consiglio Nazionale delle Ricerche (CNR), Italy

## Abstract

**Background:**

Increased body mass index is related to the incidence of thyroid cancer. However, the presentation and therapeutic outcomes of different thyroid cancers and type 2 diabetes mellitus (DM) have not been studied. This study investigated the effect of type 2 DM on the clinical presentations and therapeutic outcome of well-differentiated thyroid cancer.

**Methods and Findings:**

A retrospective analysis of adult thyroid cancer patients with or without type 2 DM admitted between January 2001 and December 2010 was performed at an institution. A total of 1,687 well-differentiated thyroid cancer patients with different histological patterns were enrolled. Among these subjects, 122 were type 2 DM patients. Patients with thyroid cancer and type 2 DM were significantly older than non-DM patients. After a mean follow-up period of 5.6±0.1 years, patients with thyroid cancer and type 2 DM showed a higher percentage of disease progression than non-DM patients (24.6% vs. 17.4%). In addition, disease-specific mortality was higher in the type 2 DM group (10.7% vs. 3.8%). Thyroid cancer patients with type 2 DM showed a higher percentage of secondary primary cancers than those without DM (10.7% vs. 4.9%). Thyroid cancer-specific survival rates in the type 2 DM and non-DM groups were 82.2% and 94.9% at 5 years, 72.9% and 91.4% at 10 years, and 36.5% and 61.3% at 20 years, respectively. Multivariate analysis showed that type 2 DM was independent of thyroid cancer-specific mortality.

**Conclusion:**

Patients with type 2 DM and well-differentiated thyroid cancer had an advanced tumor-node-metastasis stage at the time of diagnosis and an increased disease-specific mortality. Aggressive surgical procedures and close follow-up for well-differentiated thyroid cancer patients with type 2 DM are therefore necessary.

## Introduction

Cancer and diabetes mellitus (DM) as well as the associated cardiovascular complications are major health problems in developed and developing countries. Over 90% of adult patients in Taiwan diagnosed with DM have type 2 DM. The relationship between type 2 DM and cancer risk has been investigated for more than a decade. While an increased risk of cancer has been reported by some studies, others have shown a decreased risk [1], [2]. The incidence of thyroid cancer has reportedly increased in Taiwan and other developed countries over the last 10 years [3], [4]. Most well-differentiated thyroid cancers have a good prognosis, and these patients undergo long-term follow-up and treatment. In 2 recent reports, insulin resistance and obesity have been shown to increase the incidence of thyroid cancer [5], [6]; however, in another recent report, a higher body mass index (BMI) has been shown to be correlated with a lower incidence of thyroid cancer for all patients, except women >45 years of age [7]. One possible interpretation of these contradictory findings is that hyperinsulinemia in the initial stage of type 2 DM may increase cancer growth, and the anovulatory status of older women with type 2 DM may decrease the development of thyroid cancer. Previous data have illustrated that 44.3% of thyroid cancer mortality is due to non-thyroid cancer-specific mortality in Taiwan [8]. A detailed analysis of the effect of glycemia on thyroid cancer recurrence, disease-specific mortality, and other co-morbidity is therefore necessary.

The purpose of this study was to investigate the effect of type 2 DM on clinical presentations of well-differentiated thyroid cancer and on therapeutic outcome.

## Subjects and Methods

Patients with thyroid cancer and type 2 DM were identified through admission data from Chang Gung Memorial Hospital (CGMH) in Linkou, Taiwan between January 2001 and December 2010. All subjects were Chinese residents of Taiwan. Permission was obtained from the Institutional Review Board (IRB) and ethics committees of CGMH for a retrospective review of the medical records of study subjects. The IRB waived the requirement for obtaining informed consent. Confidentiality of the research subjects was maintained in accordance with the requirements of the IRB of CGMH.

Patients were ≧20 years of age and were included in this study if the indication for hospital admission was a diagnosis of thyroid cancer (code 193) and DM (code 250) in the International Classification of Disease-9 (ICD-9) clinical modification format. Type 2 DM was defined as a fasting glucose level >126 mg/dL, or a postprandial glucose level >200 mg/dL or a history of type 2 DM under treatment [9]. The clinical data was extracted as previously described [10] from the thyroid cancer database of the thyroid cancer team at the cancer center of CGMH that was established in 1994.

During the study period, 1,687 patients with well-differentiated thyroid cancers with different histological patterns were enrolled. Of these patients, 122 also had type 2 DM. All thyroid carcinomas were pathologically classified according to World Health Organization (WHO) criteria [11]. All patients were staged by International Union against Cancer-tumor-node-metastasis (UICC-TNM) criteria (6^th^ edition) [12]. Clinical postoperative progression was defined as lesions confirmed by cytology, pathology, or detectable stimulated thyroglobulin (Tg) levels (>1.2 ng/mL) for well-differentiated thyroid cancer of follicular cell origin and basal calcitonin levels above normal values (>20 pg/mL) for medullary thyroid carcinoma with any evidence suggesting a malignant lesion in imaging studies. Recurrence of thyroid cancer was defined as thyroid cancer present 1 year after thyroid surgery with or without remnant ablation with radioactive iodine (^131^I) for thyroid cancers of follicular cell origin.

Of the 122 type 2 DM patients, 18 underwent diet control only. In addition to diet control, 101 patients received oral hypoglycemic agent (OHA) treatment, and 3 received insulin therapy only. Of the 101 patients who underwent OHA treatment, 9 patients also had insulin therapy. The most commonly used OHA was metformin (72 out of 101 cases) either alone or with different sulfonylurea drugs. Other patients (29 cases) received α–glucosidase inhibitors, thiazolidinediones, or dipeptidyl peptidase IV inhibitors.

For papillary and follicular thyroid carcinomas, thyroid remnant ablation was performed 4 to 6 weeks after surgery. The ^131^I ablation dose for most patients was 1.1 GBq (30 mCi). A whole body scan (WBS) was performed 1 week after ^131^I administration using a dual-head gamma camera (Dual Genesys, ADAC, USA) equipped with high-energy collimator. A whole body image was acquired by continuous mode scanning at a speed of 5 cm/min. L-Thyroxin treatment was then initiated to reduce thyroid stimulating hormone (TSH) levels without inducing clinical thyrotoxicosis. Cases in which the foci of^ 131^I uptake extended beyond the thyroid bed were classified as persistent disease or metastases. Such patients were given increased therapeutic doses at 3.7–7.4 GBq (100 to 200 mCi); hospital isolation was arranged at doses exceeding 1.1 GBq, and a WBS was performed 2 weeks after administering the higher therapeutic dose of ^131^I. Serum Tg levels were measured using an IRMA kit (CIS Bio International, Gif Sur Yvette, France).

Admission records were surveyed for the following data: age, gender, BMI, primary tumor size, ultrasonographic findings, fine needle aspiration cytology results, thyroid function before surgery, surgical methods, histopathologic findings, TNM staging, 1-month postoperative serum Tg levels for thyroid cancer of follicular origin and calcitonin for medullary thyroid cancer, Tg antibody, diagnostic results and therapeutic ^131^I scanning, ^131^I accumulated dose, postoperative chest X-ray findings, clinical status for analysis of distant metastases via noninvasive radiological and nuclear medical study examination, treatment outcomes, causes of death, diagnosis of secondary primary cancer, and survival status. In addition, chart records were reviewed for type 2 DM patients. Data concerning DM duration, therapeutic methods, and hemoglobin A1c (HbA1c) levels were analyzed.

All data are expressed as mean ± standard error of the mean. Univariate and multivariate statistical analyses were performed to determine the significance of various factors using the Kaplan-Meier method and logistic regression [13]. Statistical significance was indicated by *p*<0.05. In addition, survival rates were calculated using the Kaplan-Meier method, and survival rates were compared using Breslow and Mantel-Cox tests.

## Results

**Figure 1:**
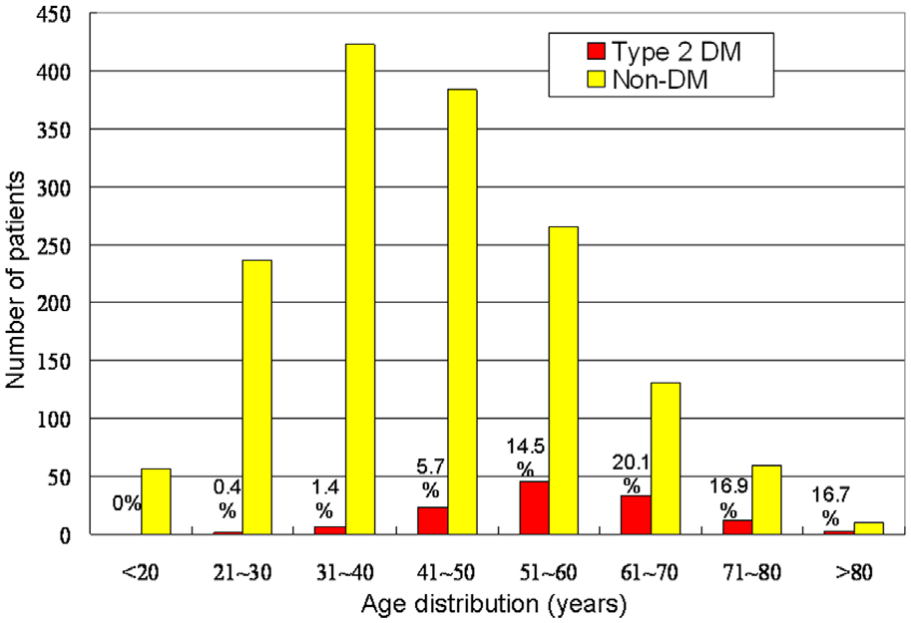
Age distribution of the subjects. Age distribution and number of thyroid cancer patients were demonstrated with and without type 2 diabetes mellitus (DM). The percentages of type 2 DM were presented in each age group.

During the 10-year study period, 1,687 patients (mean age, 44.4±0.4 years), including 122 patients (7.2%) with type 2 DM were diagnosed with thyroid cancer. There were 1,320 women and 367 men (Table 1). Of the 1,687 cases, 1,500 were papillary thyroid carcinomas, 120 were follicular thyroid cancers, 30 were Hűrthle cell carcinomas, and 37 were medullary thyroid carcinomas. Figure 1 shows the age distribution of thyroid cancer patients with and without type 2 DM.

**Table 1:** Clinical characteristics of thyroid cancer patients with and without type 2 DM.

	Type 2 DM (n = 122)	Non-DM (n = 1,565)	Total (n = 1,687)	*p* value
Age (yr)	57.6±1.0	43.4±0.4	44.4±0.4	<0.0001
Female	88 (72.1%)	1,232 (78.7%)	1,320 (78.2%)	0.0892
Total or nearly total thyroidectomy	99 (82.5%)	1,297 (82.9%)	1,396 (82.8%)	0.6266
Tumor size (cm)	2.6±0.2	2.3±0.1	2.3±0.1	0.0514
Histology type (follicular origin)	121 (99.2%)	1,529 (97.7%)	1,650 (97.8%)	0.5150
Lymph node metastases	13 (10.7%)	233 (14.9%)	246 (14.6%)	0.2020
Soft tissue invasion	28 (23.0%)	258 (16.5%)	286 (17.0%)	0.0668
Distant metastases	9 (7.4%)	80 (5.1%)	89 (5.3%)	0.2810
Multicentric	40 (32.8%)	406 (25.9%)	446 (26.4%)	0.0987
TNM stage I	44 (36.1%)	1,078 (68.9%)	1,122 (66.5%)	<0.0001
Postoperative Tg* (ng/dL)	220±66	107±11.0	116±11.6	0.0110
Cumulative ^131^I dose (mC*i*)	181±31	141±5.9	144±5.9	0.0848
Postoperative progression	30 (24.6%)	273 (17.4%)	303 (18.0%)	0.0476
Recurrence^#^	16 (13.1%)	113 (7.2%)	129 (7.6%)	0.0493
Disease-free	27 (22.0%)	462 (29.5%)	489 (29.0%)	0.0831
Secondary primary cancer	13 (10.7%)	77 (4.9%)	90 (5.3%)	0.0067
Body mass index (kg/m^2^)	26.6±0.4	24.1±0.1	24.3±0.1	<0.0001
Follow-up period (yr)	6.0±0.4	5.5±0.1	5.6±0.1	0.2626
Thyroid cancer mortality	13 (10.7%)	59 (3.8%)	72 (4.3%)	0.0003
Total mortality	15 (12.4%)	77 (4.9%)	92 (5.5%)	0.0005

Tg*: Serum thyroglobulin levels 4 to 6 weeks after thyroidectomy in papillary, follicular, and Hűrthle cell thyroid cancers. Recurrence^#^: Percent recurrence1 year after the first thyroid operation.

Thyroid cancer patients with type 2 DM were significantly older than those without DM (57.6±1.0 vs. 43.4±0.4 years; *p*<0.0001). The peak age at incidence was 51–60 years in those with concomitant type 2 DM and 31–40 years in those with thyroid cancer alone (Figure 1). The type 2 DM thyroid cancer group also had a significantly higher mean BMI than the group without DM (26.6±0.4 vs. 24.1±0.1 kg/m^2^; *p*<0.0001).

A lower percentage of patients with thyroid cancer and type 2 DM were at TNM stage I compared to those without DM (*p*<0.0001). Although a larger tumor size, more lymph node and soft tissue invasion, and more cases of distant metastases were noted in the type 2 DM group, these differences were not statistically significant. A multicentric thyroid cancer histological pattern was noted more often in the type 2 DM group, although the difference was not statistically significant (*p* = 0.0987; Table 1). During the study period, 90 (5.3%) patients were diagnosed with secondary primary cancer in addition to thyroid cancer. The most common secondary primary cancers were breast (16 cases), oropharynx (11 cases), urinary tract (7 cases), liver (7 cases), lung (7 cases), and hematologic (5 cases) (Table S1). Thyroid cancer patients with type 2 DM showed a significantly higher incidence of secondary primary cancer than patients without DM (10.7% vs. 4.9%; *p* = 0.0067).

**Figure 2:**
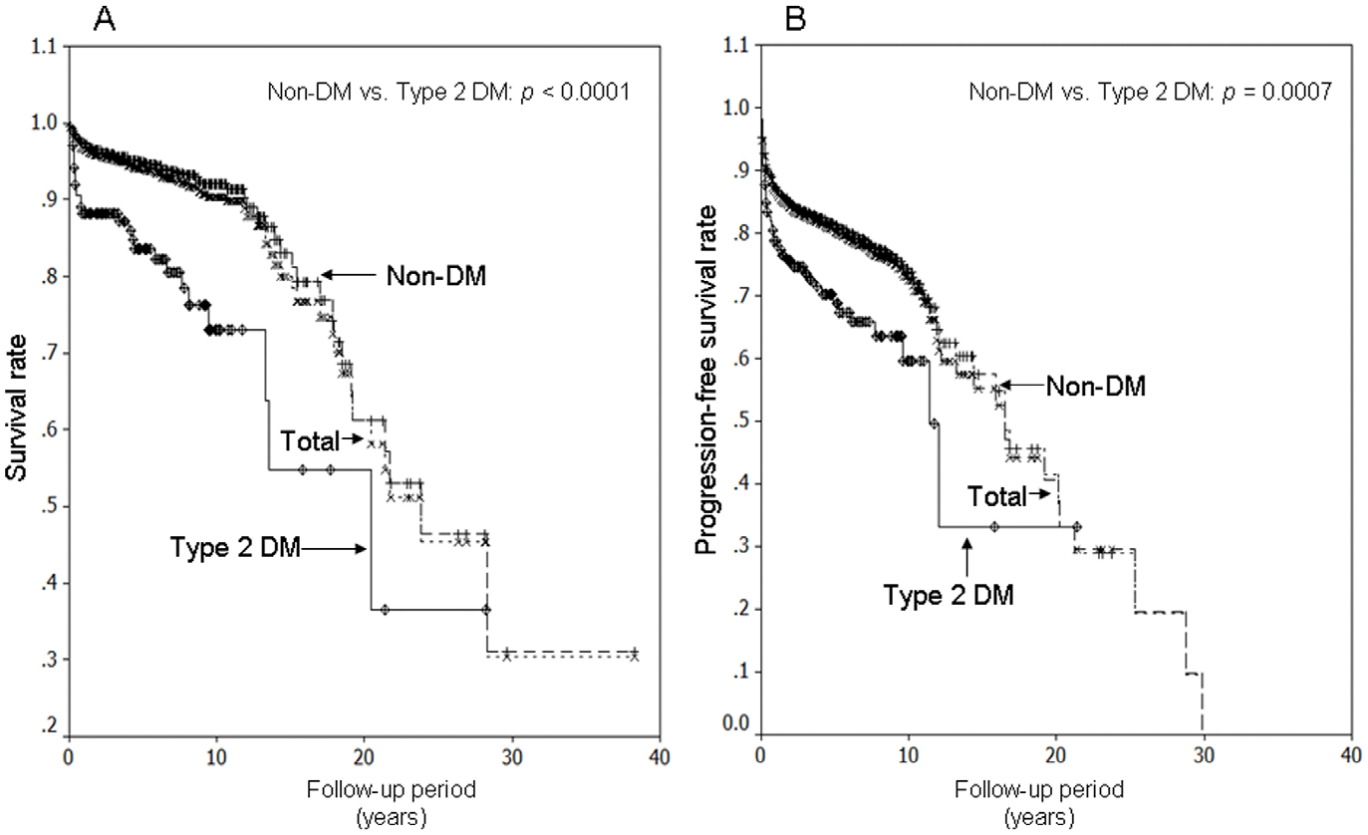
Cancer-specific and postoperative non-progressive survival curves. Cancer-specific survival curves for patients with type 2 diabetes mellitus (DM), patients without DM (non-DM), and all patients (A). Postoperative non-progressive survival rates for patients with type 2 DM, patients without DM (non-DM), and all patients (B).

After a mean follow-up period of 5.6±0.1 years, a higher percentage of disease progression was observed in thyroid cancer patients with type 2 DM compared with non-DM patients (24.6% vs. 17.4%; *p* = 0.0476). In addition, recurrence after thyroid surgery was significantly higher in the type 2 DM group. During the follow-up period, there were 72 (4.3%) deaths due to thyroid cancer and a total mortality of 92 (5.5%). Thyroid cancer-specific mortality was higher in the type 2 DM group than in the group without DM (10.7% vs. 3.8%). Figure 2A shows the thyroid cancer-specific survival curves of the type 2 DM, non-DM, and total groups. The thyroid cancer-specific survival rates in the type 2 DM, non-DM, and total groups were 82.2%, 94.9%, and 94.0% at 5 years; 72.9%, 91.4%, and 90.5% at 10 years; and 36.5%, 61.3%, and 61.3% at 20 years, respectively. The postoperative progression-free survival rates for the type 2 DM, non-DM, and total groups were 68.6%, 81.2%, and 80.2% at 5 years; 59.2%, 73.7%, and 72.5% at 10 years; and 33.1%, 37.3%, and 36.9% at 20 years, respectively (Figure 2B). A statistically significant difference was found between type 2 DM and non-DM groups with respect to thyroid cancer specific survival and progression-free survival (*p*<0.05).

The mean HbA1c levels of the 122 type 2 DM patients with thyroid cancer was 8.0±0.2% at the time of thyroid operation. A comparison of postoperative progression-free survival and progressive disease in patients with type 2 DM and thyroid cancer is shown in Table 2. Tumor size, soft tissue invasion, distant metastases, TNM stage, and postoperative Tg levels were significantly related to progression. The operative method and mean HbA1c levels did not differ between the progressive and progression-free groups. In total, 82.8% of type 2 DM patients underwent OHA treatment (101 out of 122 cases), and 72 patients were treated with metformin. Although metformin therapy led to higher percentages of progression-free survival and overall survival compared with progressive disease and mortality, no statistically significant differences were observed (Tables 2 and 3).

**Table 2:** Clinical characteristics of of thyroid cancer patients with type 2 DM who exhibited postoperative progression-free survival or progressive disease.

	Progression-free (n = 92)	Progressive disease (n = 30)	*p* value
Age (yr)	56.7±1.1	60.0±1.8	0.1472
Female	67 (72.8%)	21 (70%)	0.7643
Total or nearly total thyroidectomy	76 (82.6%)	23 (76.7%)	0.4699
Tumor size (cm)	2.2±0.2	4.0±0.4	<0.0001
Lymph node metastases	11 (12.0%)	2 (15.4%)	0.5167
Soft-tissue invasion	15 (16.3%)	13 (43.3%)	0.0002
Distant metastases	0	9 (30.0%)	<0.0001
Multicentric	32 (34.8%)	8 (26.7%)	0.5045
TNM stage I	43 (46.7%)	1 (3.3%)	<0.0001
Postoperative Tg * (ng/dL)	24.2±11.6	771±223	<0.0001
Cumulative ^131^I dose (mC*i*)	75.4±8.7	461±89	<0.0001
Secondary primary cancer	9 (9.8%)	4 (13.3%)	0.7335
HbA1c (%)	8.1±0.2	7.8±0.3	0.5333
Follow-up period (yr)	5.3±0.4	8.1±0.1	0.0038
Thyroid cancer mortality	0	13 (43.3%)	<0.0001
Total mortality	2 (2.2%)	13 (43.3%)	<0.0001
Body mass index (kg/m^2^)	26.7±0.5	26.2±0.9	0.6434
DM treatment			
Diet alone (18)	13 (14.1%)	5 (16.6%)	0.8783
OHÂ (101)	77 (83.7%)	24 (80.0%)	
Insulin alone (3)	2 (2.2%)	1 (3.3%)	
OHA with metformin treatment	56 (60.9%)	16 (53.3%)	0.5242

Tg*: Serum thyroglobulin levels 4 to 6 weeks after thyroidectomy in papillary, follicular and Hűrthle cell thyroid cancers. OHÂ: Treatment with an oral hypoglycemia agent, including 9 cases of combination treatment with insulin.

**Table 3:** Clinical characteristics of survival and mortality groups of thyroid cancer patients with type 2 DM.

	Survival (n = 109)	Mortality (n = 13)	*p* value
Age (yr)	57.3±1.0	59.6±2.8	0.4544
Female	80 (73.4%)	8 (61.5%)	0.3494
Total or nearly total thyroidectomy	89 (81.7%)	10 (76.9)	0.7096
Tumor size (cm)	2.4±0.2	4.9±0.6	<0.0001
Lymph node metastases	12 (11.0%)	1 (7.7%)	>0.9999
Soft tissue invasion	22 (20.2%)	6 (46.2%)	0.0353
Distant metastases	4 (3.7%)	5 (38.5%)	0.0006
Multicentric	38 (34.9%)	2 (15.4%)	0.1573
TNM stage I	44 (40.4%)	0	0.0040
Postoperative Tg* (ng/dL)	110±45.5	1,135±405	<0.0001
Cumulative ^131^I dose (mC*i*)	123±19.0	657±180	<0.0001
Secondary primary cancer	12 (11.0%)	1 (7.7%)	>0.9999
HbA1c (%)	8.1±0.2	7.1±0.3	0.1796
Follow-up period (yr)	5.8±0.4	7.2±1.6	0.3124
Body mass index (kg/m^2^)	26.8±0.4	24.4±0.1	0.1386
DM treatment			
Diet alone (18)	15 (13.8%)	3 (23.1%)	0.2689
OHÂ (101)	92 (84.4%)	9 (69.2%)	
Insulin alone (3)	2 (1.8%)	1 (7.7%)	
OHA with metformin treatment	66 (60.6%)	6 (46.2%)	0.3775

Tg*: Serum thyroglobulin levels 4 to 6 weeks after thyroidectomy in papillary, follicular and Hűrthle cell thyroid cancers. OHÂ: Treatment with an oral hypoglycemia agent, including 9 cases of combination treatment with insulin.

A comparison of thyroid cancer mortality with non-thyroid cancer mortality is shown in Table 3. Male gender, soft tissue invasion, distant metastases, and TNM stage were significantly related to cancer mortality. An analysis of other factors using logistic regression to avoid the influence of age on thyroid cancer mortality is shown in Table 4. Independent factors associated with thyroid cancer-specific mortality were age and type 2 DM. Mortality in the type 2 DM group was 4.3 times greater than that in the non-DM group.

**Table 4:** Multivariate analysis of different parameters related to the mortality of thyroid cancer patients with and without type 2 DM using Cox proportional hazard model.

	Patient number = 1,687			
	*p* value.	Hazards ratio	95% CI	
			Lower	Upper
Age at diagnosis (yr)	0.0002	1.117	1.054	1.185
Gender	0.8037	1.150	0.382	3.463
Type 2 DM	0.0230	4.301	1.223	15.129
Tumor size	0.0830	1.124	0.985	1.282
Postoperative Tg* (ng/mL)	0.0175	1.001	1.000	1.001
Postoperative ^131^I uptake (%)	0.0272	0.954	0.915	0.995
Multicentric	0.0655	0.217	0.043	1.103
TNM stage	0.0498	2.230	1.001	4.971

Tg*: Serum thyroglobulin levels 4 to 6 weeks after thyroidectomy in papillary, follicular and Hűrthle cell thyroid cancer.

## Discussion

Hyperinsulinemia is a common characteristic of obesity, metabolic syndrome and type 2 DM. A recent large European prospective case-control study reported a moderate positive association between obesity, height, and differentiated thyroid carcinoma [14]. Type 2 DM was less often reported in patients with thyroid nodules or cancer [15], [16]. In the current study, 7.2% of well-differentiated thyroid cancer patients with a mean age of 44.4 years had type 2 DM, which is similar to the incidence of type 2 DM in the general population in our area [17]. Although type 2 DM patients may have a greater chance of being examined for thyroid nodules, they exhibited a larger tumor size and a more advanced TNM stage at the time of thyroid operation than patients without DM.

Most well-differentiated thyroid cancer patients have a long follow-up period and a good prognosis [18]. In addition, in the current study, thyroid cancer patients were treated at the same institute and usually underwent consistent surgical and postoperative adjuvant therapy. During the follow-up period for patients with type 2 DM and well-differentiated thyroid cancer, the growth promotion effects of hyperinsulinemia may worsen the prognosis. Although the plasma insulin level was not determined in our study, the fact that the type 2 DM group had a higher BMI than the non-DM group was indirect evidence of relative hyperinsulinemia. Postoperative progression was more frequent in the type 2 DM group than in the non-DM group. Moreover, in patients with postoperative progression, recurrence diagnosed 1 year after the thyroid operation was much more frequent in the type 2 DM group than in the non-DM group.

In the current study, an increased secondary primary cancer rate was observed in the thyroid cancer with type 2 DM group compared with the thyroid cancer without DM group. Breast cancer is the leading female cancer in Taiwan and many other developed countries. The long-term survival and hyperinsulinemia status of the patients with well-differentiated thyroid cancer and type 2 DM may be one of the major causes of a higher incidence of secondary primary cancer. A previous study indicated that mortality from site-specific malignancies was worse in patients with type 2 DM than in the general population [19]. In this study, the total mortality of thyroid cancer patients was increased when type 2 DM was also presented. Age and gender are important factors for the interpretation of prognosis or cancer mortality in well-differentiated thyroid cancer [20]. In addition, the postmenopausal status of female patients is another factor that may influence long-term outcomes [21]. However, our regression analysis indicated that type 2 DM in thyroid cancer patients resulted in high cancer-related mortality.

A recent report concerning metformin treatment showed a significant decrease in the size of thyroid nodules in patients with insulin resistance and small thyroid nodules [22]. Theoretically, diabetes treatments involving insulin or metformin may increase or decrease hyperinsulinemia, and these changes may influence the incidence or treatment results in different cancers [23], [24]. In this study, which included a limited series of thyroid cancer with type 2 DM cases, metformin treatment lessened disease progression and improved survival; however, the data was not statistically significant. The duration of hyperinsulinemia, oncogenetic and environmental factors, and different therapeutic modalities may influence the treatment outcomes.

In conclusion, patients with well-differentiated thyroid cancer and type 2 DM were at more advanced TNM stages at the time of primary operation and experienced increased thyroid cancer-specific mortality and recurrence rates than patients without DM. Therefore, more aggressive surgical treatment followed by adjuvant ^131^I remnant ablation and therapy is recommended for well-differentiated thyroid cancer patients with type 2 DM. In addition, periodic screening for secondary primary cancer at the time of follow-up should be performed.

## Supporting Information

Table S1 Secondary primary cancer in thyroid cancer patients.
(DOC)
