# Supplementary material for: Disease-Specific Mortality and Secondary Primary Cancer in Well-Differentiated Thyroid Cancer with Type 2 Diabetes Mellitus
Source: PLoS One. 2013 Jan 31;8(1):e55179. doi: 10.1371/journal.pone.0055179 (PMC3561360; doi:10.1371/journal.pone.0055179)
Supplement: Table S1 — Secondary primary cancer in thyroid cancer patients. (DOC) [file pone.0055179.s001.doc]

**Table S1:** Secondary primary cancer in thyroid cancer patients.

| **Cancers (N)** | **Type 2 DM (13)** | **Non-DM (77)** | **Total (90)** |
| --- | --- | --- | --- |
| Breast (F) | 2 | 14 | 16 |
| Oropharynx | 5 | 7 | 12 |
| Liver | 0 | 7 | 7 |
| Urinary tract | 2 | 5 | 7 |
| Lung | 0 | 7 | 7 |
| Hematologic | 1 | 4 | 5 |
| Colon, rectal | 1 | 4 | 5 |
| NPC* | 0 | 4 | 4 |
| Gastric | 0 | 3 | 3 |
| Prostate (M) | 0 | 3 | 3 |
| Cervical (F) | 0 | 3 | 3 |
| Ovary (F) | 0 | 3 | 3 |
| Bone sarcoma | 0 | 2 | 2 |
| Others | 2 | 11 | 13 |
| Total cancer | 13 | 77 | 90 |

F: females; NPC* nasopharyngeal cancer; M: males
